# Supplementary material for: A small molecule that induces translational readthrough of CFTR nonsense mutations by eRF1 depletion
Source: Nat Commun. 2021 Jul 16;12:4358. doi: 10.1038/s41467-021-24575-x (PMC8285393; doi:10.1038/s41467-021-24575-x)
Supplement: Supplementary file 1 — Supplementary Information [file 41467_2021_24575_MOESM1_ESM.pdf]

SUPPLEMENTARY INFORMATION

Supplementary Notes:

Information related to Suppl. Fig. 4: CFTR modulators augment CFTR ion channel function following readthrough combination therapy across multiple *CFTR* nonsense mutations.

The use of multiple CFTR modulator drugs that act on different stages of CFTR synthesis and maturation to restore channel function are highly effective for the common F508del mutation [1-3]. Recently, it was shown that the function of CFTR protein variants generated by readthrough could also be augmented by the CFTR potentiator ivacaftor (formerly VX-770) and corrector lumacaftor (formerly VX-809) [1,3]. To maximize the functional rescue of CFTR generated by the readthrough of the G542X nonsense mutation, we included lumacaftor and ivacaftor in the CFTR functional assays. First, we assessed CFTR function in FRT cells stably expressing a human *CFTR*-G542X cDNA and treated with SRI-37240, G418, and lumacaftor for 48 hours, followed by the acute addition of ivacaftor during current assays. This combination significantly improved CFTR-dependent chloride conductance, reaching 14% of the wild-type control (Suppl. Fig. 4A-B). Six percent (6%) of wild-type function was observed when lumacaftor was omitted (SRI-37240+G418) and 1.6% of wild-type function was achieved when lumacaftor and ivacaftor were used with a single readthrough agent (Suppl. Fig. 4A-B). Western blotting demonstrated that lumacaftor treatment increased the CFTR band C to band B ratio, which is consistent with the more fully glycosylated full-length CFTR and an indicator of cellular processing efficiency (Suppl. Fig. 4C).

Since the efficiency of CFTR nonsense suppression is sensitive to the mRNA context surrounding a PTC [4], we also evaluated the impact of SRI-37240 and G418 on other common *CFTR* PTC contexts with a UGA stop codon (R553X, R1162X and W1282X) in FRT cells, each with lumacaftor and ivacaftor to augment CFTR rescue (Suppl. Fig. 4D-I). As with G542X, the R553X and R1162X *CFTR* mutations responded to SRI-37240, but readthrough was enhanced when co-administered with G418 (Suppl. Fig. D-G); lumacaftor further augmented this effect. SRI-37240, G418, and lumacaftor pretreatment augmented CFTR-dependent G_t to 8-14% of wild-type in FRT cells expressing the R553X (UGAG) and R1162X (UGAG) constructs, respectively, after full activation by forskolin and ivacaftor. The truncated CFTR protein that is formed as a result of the W1282X (UGAA) mutation exhibits residual function even in the truncated state and is highly responsive to pharmacologic correction and potentiation under high expression conditions [3]; thus, its response profile differed. SRI-37240 strongly augmented CFTR-dependent chloride conductance as monotherapy (9% of wild-type), which could be enhanced to 19% by lumacaftor and ivacaftor. However, the further addition of G418 did not substantially improve CFTR function from the W1282X allele as compared to SRI-37240 alone. However, combination therapy with SRI-37240, G418, and lumacaftor rescued 24% of wild-type CFTR activity with potentiation with ivacaftor, which was superior to the SRI-37240 and G418 dual treatment (16% of wild type; Suppl. Fig. 4H-I). Taken together, these results indicate that SRI-37240 can rescue CFTR function across multiple *CFTR* nonsense mutations, but readthrough efficacy for most *CFTR* nonsense mutations was substantially improved by the addition of G418 and by

CFTR modulator compounds, which augmented the function of the readthrough product.

Information related to Suppl. Fig. 8: Off-target effects of SRI-37240 and SRI-41315 on other ion channels

SRI-37240, and to a lesser extent SRI-41315, significantly reduced baseline and amiloride-sensitive Isc of the epithelial sodium chloride channel (ENaC) when administered for 24 hours (Suppl. Fig. 8A). Acute addition of SRI-37240 on primary HBE cells homozygous for the F508del mutation demonstrated the effect was immediate (Suppl. Fig. 8B), but only when added to the apical compartment and not the basolateral side (Suppl. Fig. 8C). CFTR currents in wild-type CFTR expressing cells were also slightly reduced with acute addition of SR-37240 (Suppl. Fig. 8D). Taken together, these data show that SRI-41315 and SRI-37240 enhanced CFTR activity by inducing translational readthrough of nonsense mutations when co-administered with the aminoglycoside G418, but are not suitable as developmental candidates for cystic fibrosis due to their relatively low efficacy in HBE cells as compared to therapeutic thresholds established for other CFTR modulators [2] and their undesirable effects on apical ion transporters that may diminish rescue of CFTR function.

Supplementary Table 1. Control Data Summary

Average Z'	0.78	$Z' = 1 - 3(StdDev_{PosCtrl} + StdDev_{NegCtrl}) / (Mean_{PosCtrl} - Mean_{NegCtrl})$
Average Pos Ctrl CV	6.8	$\%CV = 100 * StdDev_{PosCtrl} / Mean_{NegCtrl}$
Average S/B	37	$S/B = Mean_{PosCtrl} / Mean_{NegCtrl}$
Average S/N	15	$S/N = (Mean_{PosCtrl} - Mean_{NegCtrl})/sqrt(StdDev_{PosCtrl\hat{}2} + StdDev_{NegCtrl\hat{}2})$

Supplementary Table 2: Oligos used in this study.

Oligo Name / Sequence	Purpose
DB4078:<br>5'-TCGAGCCAAGCTTGCATGCCTGCAGGT CGACTCTAGAGGATCCCCGGGGAATTC GC-3'<br>DB4079:<br>5'-GGCCGCGAATTCCCCGGGGATCCTCTA GAGTCGACCTGCAGGCATGCAAGCTTGGC-3'	Primer set used to introduce a new multi-cloning site into the pFN(Nluc_CMV_Neo) plasmid.
DB4144:<br>5'-GGGACCCTGTGAAACGGCAAC-3'<br>DB4145:<br>5'-GTTGCCGTTTCACAGGGTCCC-3'	Primer set used to introduce a point mutation into pDB1333.
1. CFTR: primer set ID<br>Hs00357011_m1<br><br>*2. GAPDH* primer set ID:<br>Rn01775763_g1, Hs02786624_g1<br><br>*3. TBP* primer set ID:<br>Hs.PT.58.20792004<br><br>*4. eRF1* primer set ID:<br>Hs1107363_gH<br><br>5. NanoLuc primer set:<br>DB4126: 5'-ATTGTCCTGAGCGGTGAAA-3'<br>DB4127: 5'-CACAGGGTACACCACCTTAAA-3'<br><br>6. Rat *GAPDH* primer set:<br>DB4187: 5'-AGTCAAGGCTGAGAATGGGAAG-3'<br>DB4188: 5’-GGTGGTGAAGACGCCAGTAGA-3'<br><br>7. Rat beta-actin set:<br>DB4365: 5'-CAACTGGGACGATATGGAGAAG-3'<br>DB4366: 5'-CTCGAAGTCTAGGGCAACATAG-3'<br><br>8. Rat alpha-1-tubulin set:<br>DB4183: 5'-CAACACCTTCTTCAGTGAGACAG G-3'<br>DB4184: 5'-TCAATGATCTCCTTGCCAATGGT-3'	Primers to perform quantitative real-time PCR; Primer sets 1-4 purchased from Life Tech.

Supplementary Figure 1

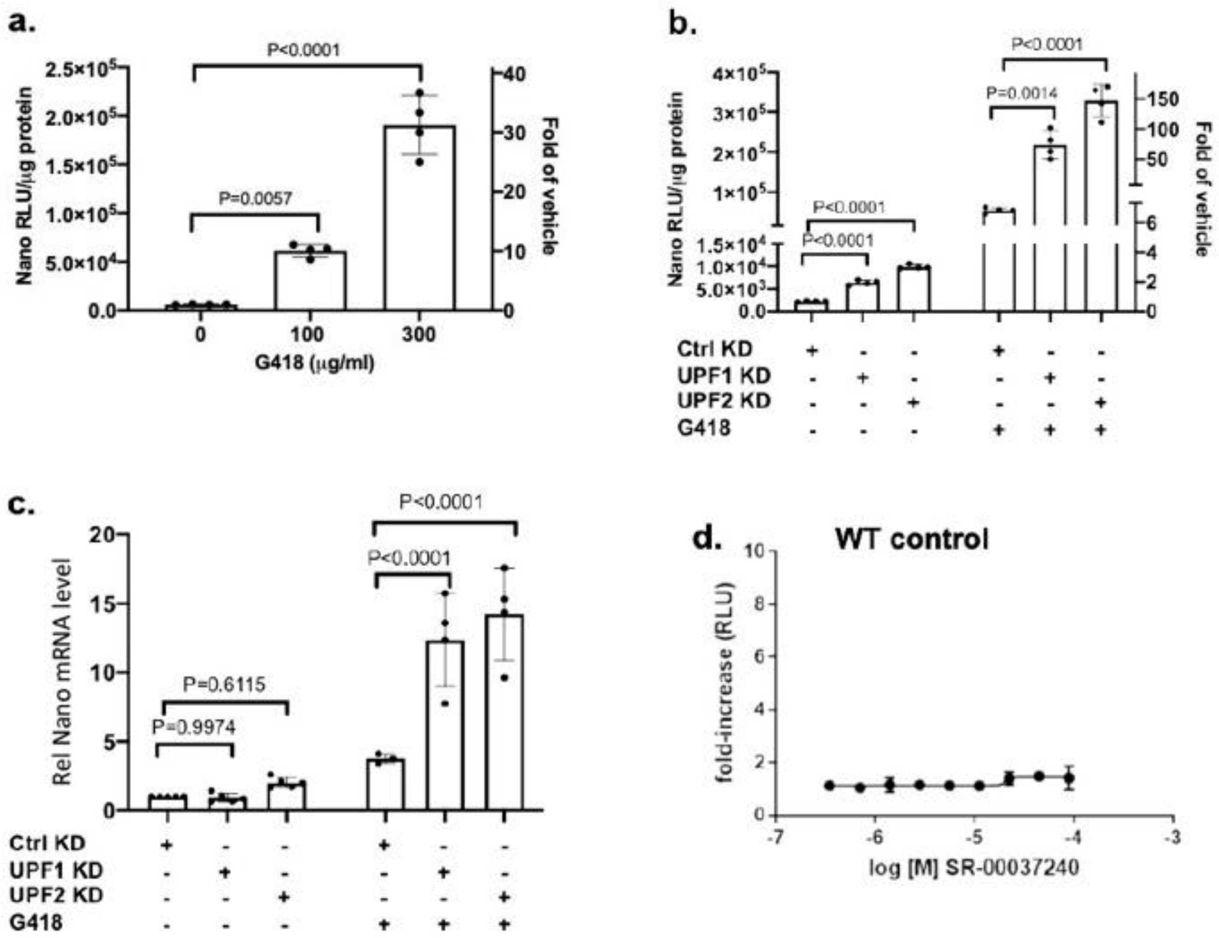

Suppl. Fig. 1: Characterization of the W134X NanoLuc dual RT/NMD reporter in FRT cells. a. Reporter NanoLuc activity in response to G418. **b.** Reporter NanoLuc activity in response to NMD inhibition *via* knockdown of UPF1 or UPF2 +/- 300 µg/mL G418. **c.** Quantitation of NanoLuc mRNA abundance in reporter cells subjected to UPF1 or UPF2 knockdown +/- 300 µg/mL G418. **d.** Dose response of a WT NanoLuc reporter (no PTC present) to SRI-37240 in FRT cells. Fold-changes in NanoLuc activity and mRNA are relative to the values obtained from untreated cells or cells transfected with a negative knockdown control, respectively. NanoLuc activity (RLU/µg protein) in panels a, b, and d is expressed as the mean +/- SD of a representative experiment performed in quadruplicate (n=4). Replicate experiments generated similar results. mRNA abundance is expressed as the mean +/- SEM of NanoLuc mRNA normalized to three different controls (*GAPDH*, *ACTB* and *TUBA1B*) from two biological samples, each performed in quadruplicate. For panels a-c, the exact p values comparing the cohorts indicated by the brackets were calculated using 2-way ANOVA.

Supplementary Figure 2

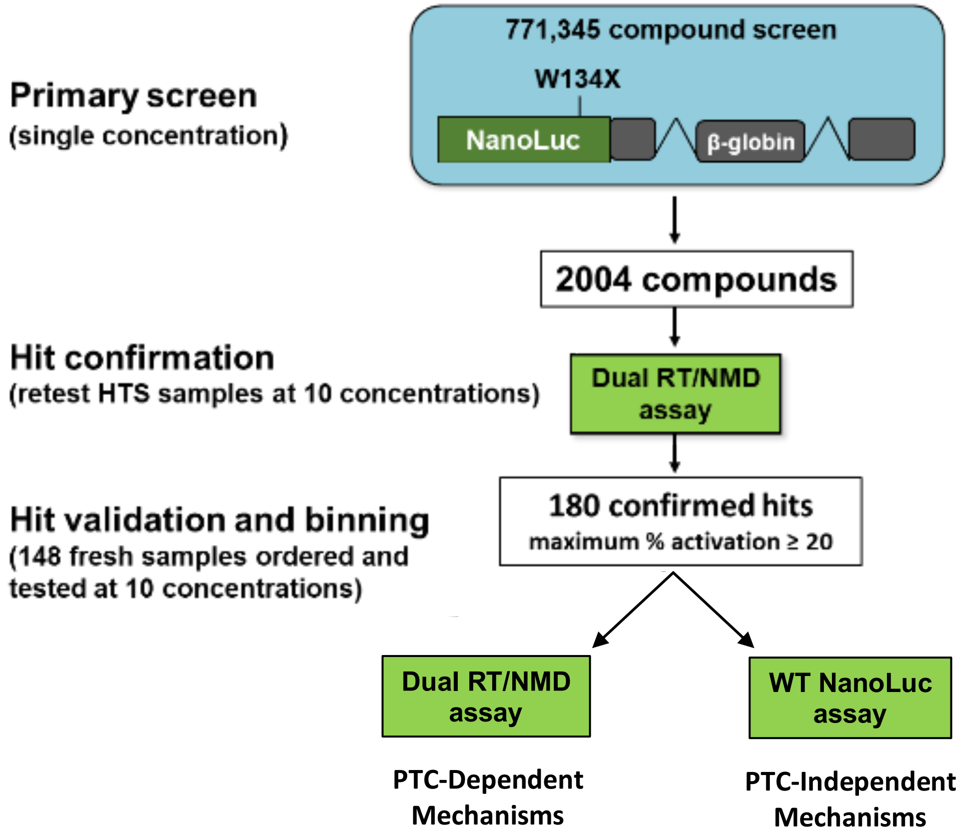

Suppl. Fig. 2: Compound progression pathway. High throughput screening strategy used to identify nonsense suppression compounds.

Supplementary Figure 3

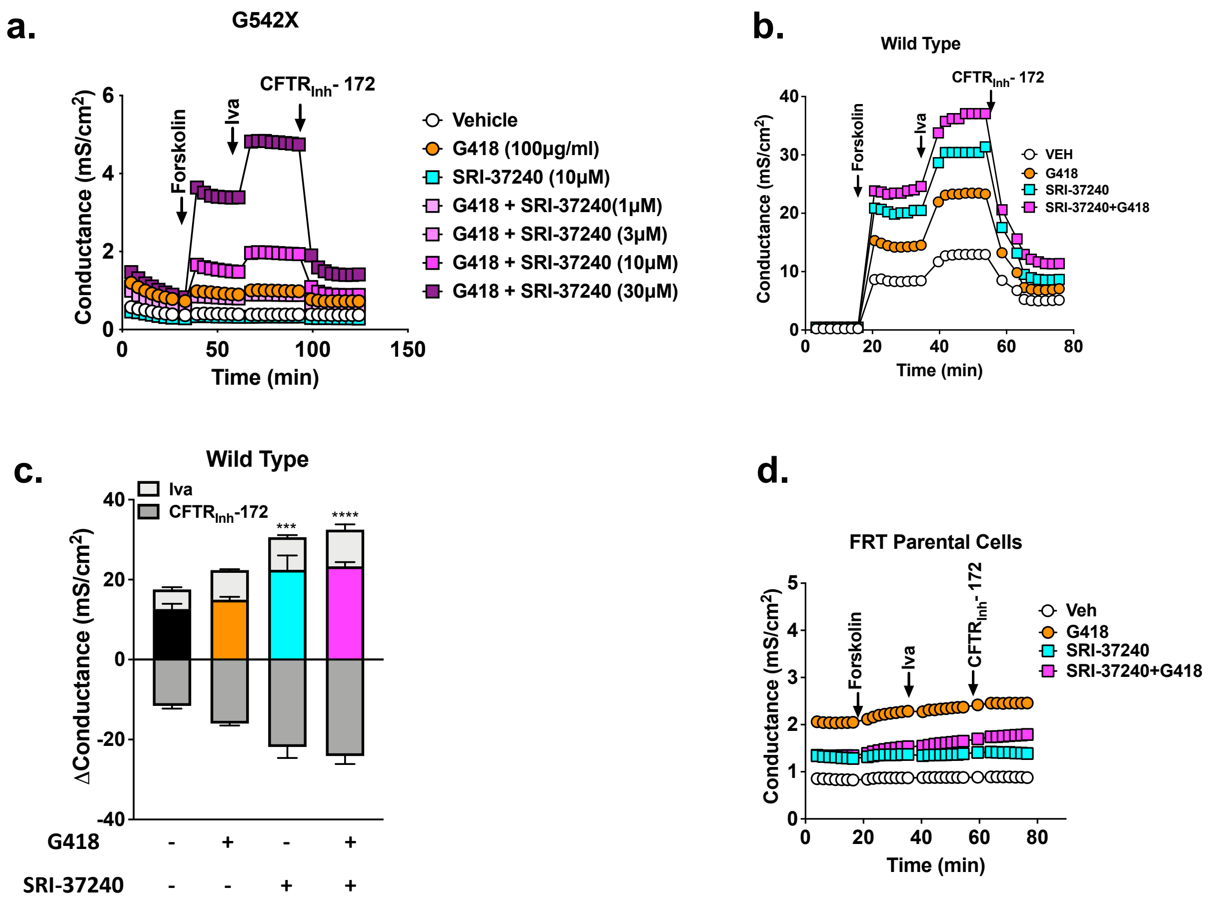

Suppl. Fig. 3: CFTR expression and function in FRT cells stably transduced with wild-type or G542X *CFTR*. FRT cells were grown in liquid-liquid interface followed by a 48-hour treatment with SRI-37240 (10 µM, unless otherwise indicated) +/- G418 (100 µg/ml). **a.** Representative conductance tracing in *CFTR*-G542X

FRT cells treated with G418 alone or with increasing concentrations of SRI-37240. CFTR activity was measured as the change from baseline conductance following activation by the addition of forskolin (10 µM) and ivacaftor (10 µM), and also after inhibition with $CFTR_{Inh}$-172 (10 µM). **b.** Representative conductance tracings in isogenic wild-type FRT cells after treatment with SRI-37240 and G418 alone or in combination. **c.** Corresponding summary of change in conductance after forskolin and ivacaftor induced CFTR conductance and $CFTR_{Inh}$-172 mediated inhibition (n=3, Data are statistically analyzed using Two-way ANOVA followed by Tukey's post-hoc test, ***p=0.0002 (Vehicle vs SRI-37240), ****p<0.0001 (Vehicle vs SRI-37240 + G418). **d.** Representative conductance tracings in parental FRT cells (without CFTR expression) after treatment with SRI-37240 and G418 alone or in combination. Each experiment was performed 3-4 times with n=2-4 monolayers per condition each replicate.

Supplementary Figure 4

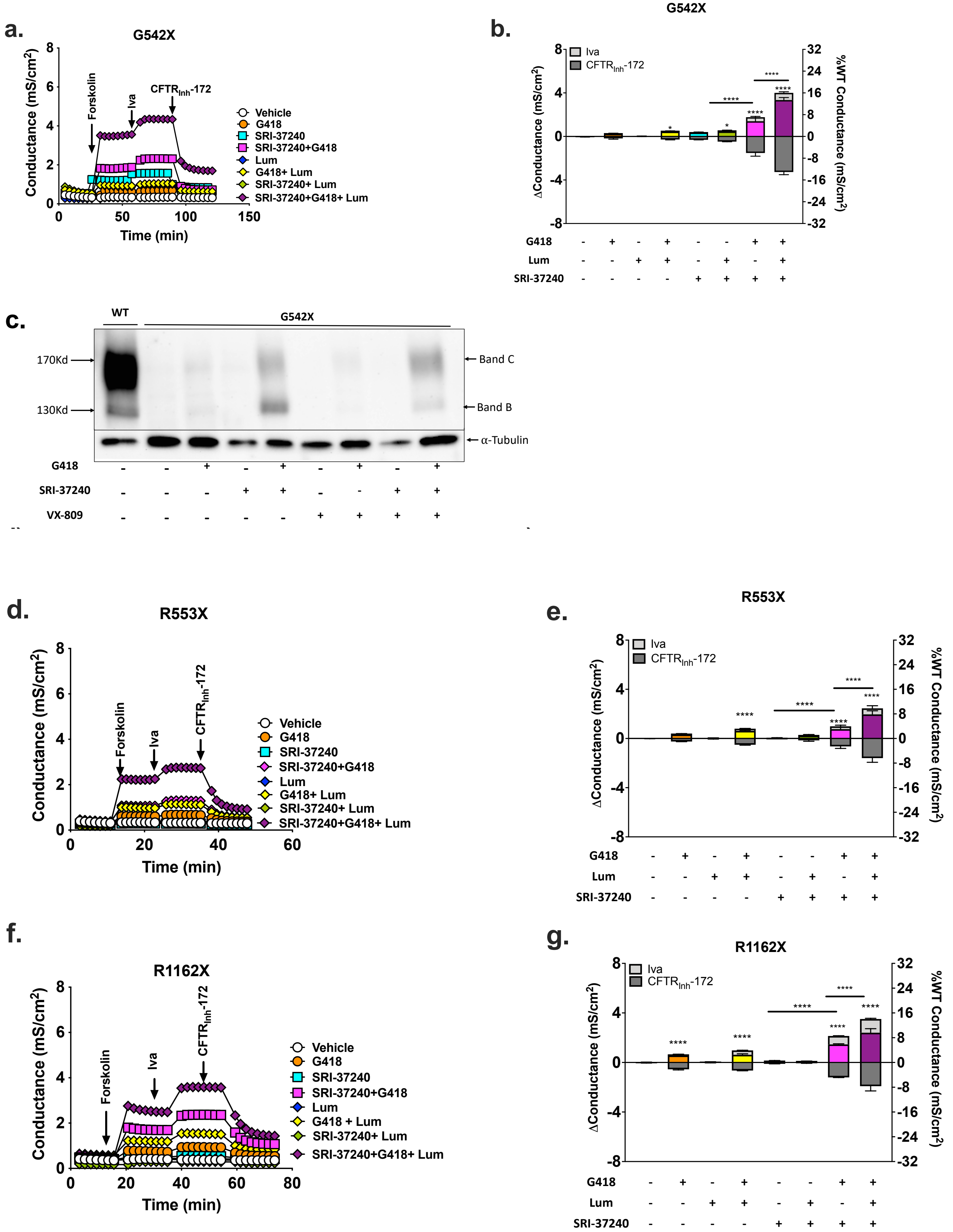

Supplementary Figure 4 continued

h.

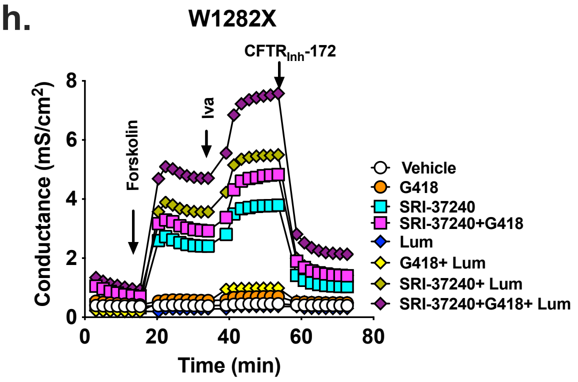

i.

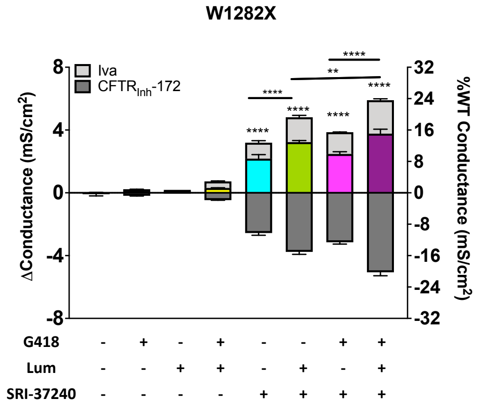

Suppl. Fig. 4: CFTR modulators augment CFTR ion channel function following readthrough combination therapy across multiple *CFTR* nonsense mutations. Representative CFTR-specific conductance tracings and summary data of FRT cells stably transduced with various *CFTR* nonsense mutations and then treated with SRI-37240 (10 μM) alone or in combination with G418 (100 μg/ml) and/or lumacaftor (VX-809, 3 μM) for 48 hours at liquid-liquid interface. CFTR function was measured as the change from baseline conductance following the addition of forskolin (10 μM) and ivacaftor (10 μM) and then confirmed by inhibition with $CFTR_{Inh}$-172 (10 μM): data are also expressed as % of FRT wild type CFTR conductance in isogenic FRT cells. **a-b.** Representative conductance and quantitation, respectively, in FRT cells expressing *CFTR* -G542X (n=3, data are statistically analyzed using Two-way ANOVA followed by Tukey's post-hoc test, p=0.028 (Vehicle vs G418+Lum), *p=0.020 (Vehicle vs SRI-37240 + Lum), ****p<0.0001 for all other comparisons shown. **c.** Corresponding CFTR western blot in *CFTR*-G542X FRT cells (wild type - 5μg, G542X - 30 μg). Representative conductance and quantitation, respectively, in FRT cells expressing: **d, e.** *CFTR*-R553X (n=3, data are statistically analyzed using Two-way ANOVA followed by Tukey's post-hoc test, ****p<0.0001 for all comparisons shown); **f, g.** *CFTR*-R1162X (n=3, data are statistically analyzed using Two-way ANOVA followed by Tukey's post-hoc test, ****p<0.0001 for all comparisons shown); **h, i.** *CFTR*-W1282X. (n=3, data are statistically analyzed using Two-way ANOVA followed by Tukey's post-hoc test, **p<0.003 (SRI-37240+Lum vs SRI-37240+G418+Lum, ****p<0.0001 for all other comparisons shown).

Supplementary Figure 5

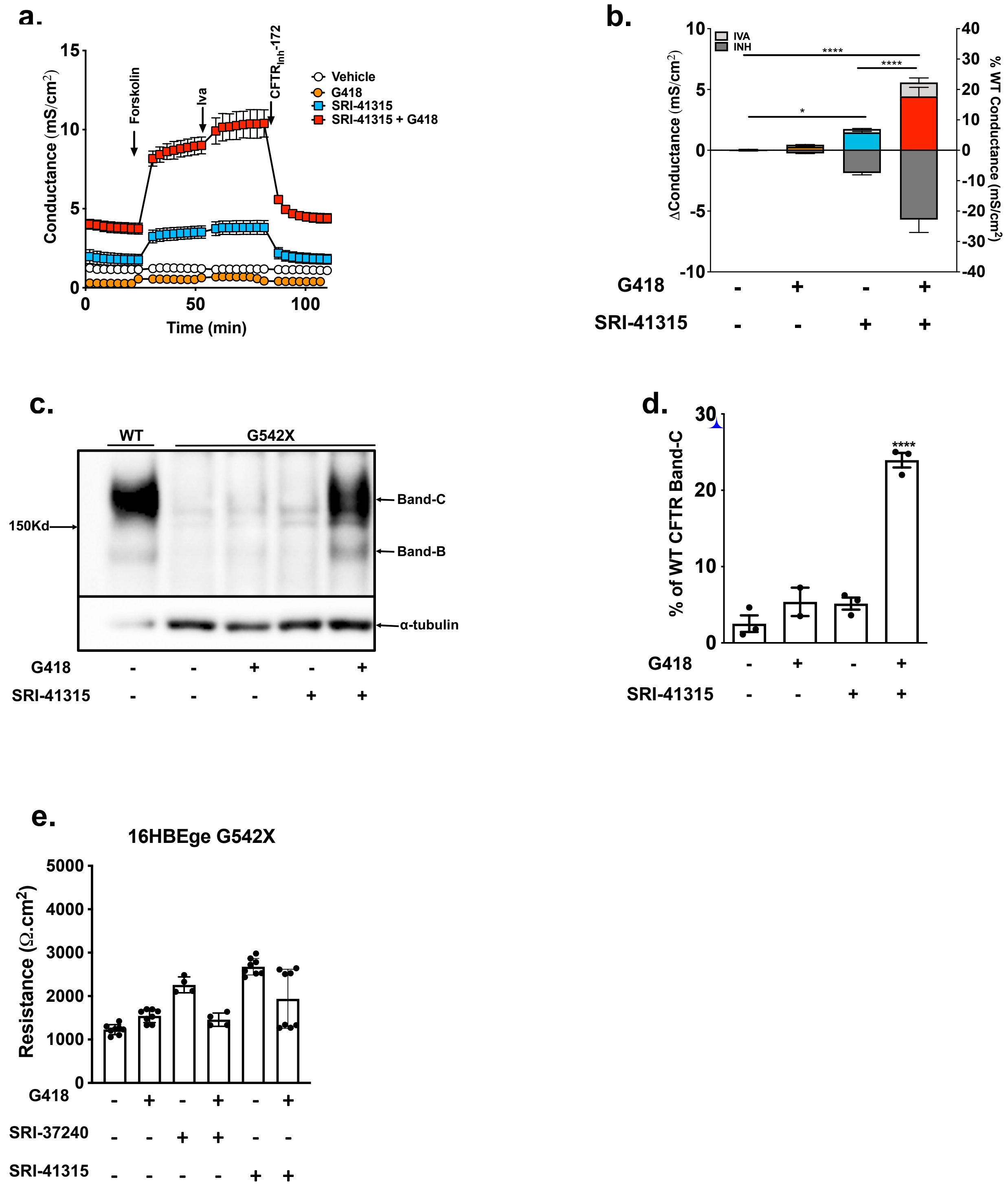

Suppl. Fig. 5: Enhanced CFTR expression and function in *CFTR*-G542X FRT and 16HBEge cells after combination treatment with SRI-41315 and G418. *CFTR*-G542X FRT cells were grown on permeable supports and then treated at the liquid-liquid interface with SRI-41315 (1 µM) alone or in combination with G418 (100 µg/ml) for 48 hours. CFTR activity was then assayed and compared to the vehicle control. **a.** Representative conductance tracings after acute addition of forskolin and ivacaftor (10 µM) to activate CFTR function followed by the addition of $CFTR_{Inh}$-172 (10 µM) to inhibit CFTR function. **b.** Corresponding summary data after forskolin and ivacaftor induced CFTR conductance and $CFTR_{Inh}$-172 inhibited CFTR conductance; data also expressed as % of wild-type CFTR conductance in isogenic FRT cells (i.e. 25 mS/cm^2) (n=3, data are expressed as mean ± S.D. and statistically analyzed using Two-way ANOVA followed by Tukey's post-hoc test, *p=0.0182 (Vehicle vs SRI-41315), ****p<0.0001 for all other comparisons shown. **c.** Representative western blot showing the abundance of full-length CFTR band C (fully processed) and band B (unprocessed) after combination treatment compared to single agent treatments. The wild-type control loaded was 30% of the total protein loaded for the G542X lysates. **d.** Corresponding western quantitation of CFTR band C (relative to tubulin) (n=3, data are expressed as mean ± S.D. and statistically analyzed using Ordinary One-way ANOVA followed by Dunnett's post-hoc test, ****p<0.0001 (Vehicle vs SRI-41315+G418). **e.** Baseline transepithelial resistances of *CFTR*-G542X 16HBEge cells treated with SRI-37240 (10 µM), SRI-41315 (5 µM), or G418 (100 µg/ml) alone or combinations (functional data shown in Fig. 5a-b). *p≤0.05,

****$p \leq 0.0001$. Each experiment was performed 3-4 times with n=2-4 monolayers per condition during each replicate. 3-4 repeats for each western blot experiment.

Supplementary Figure 6

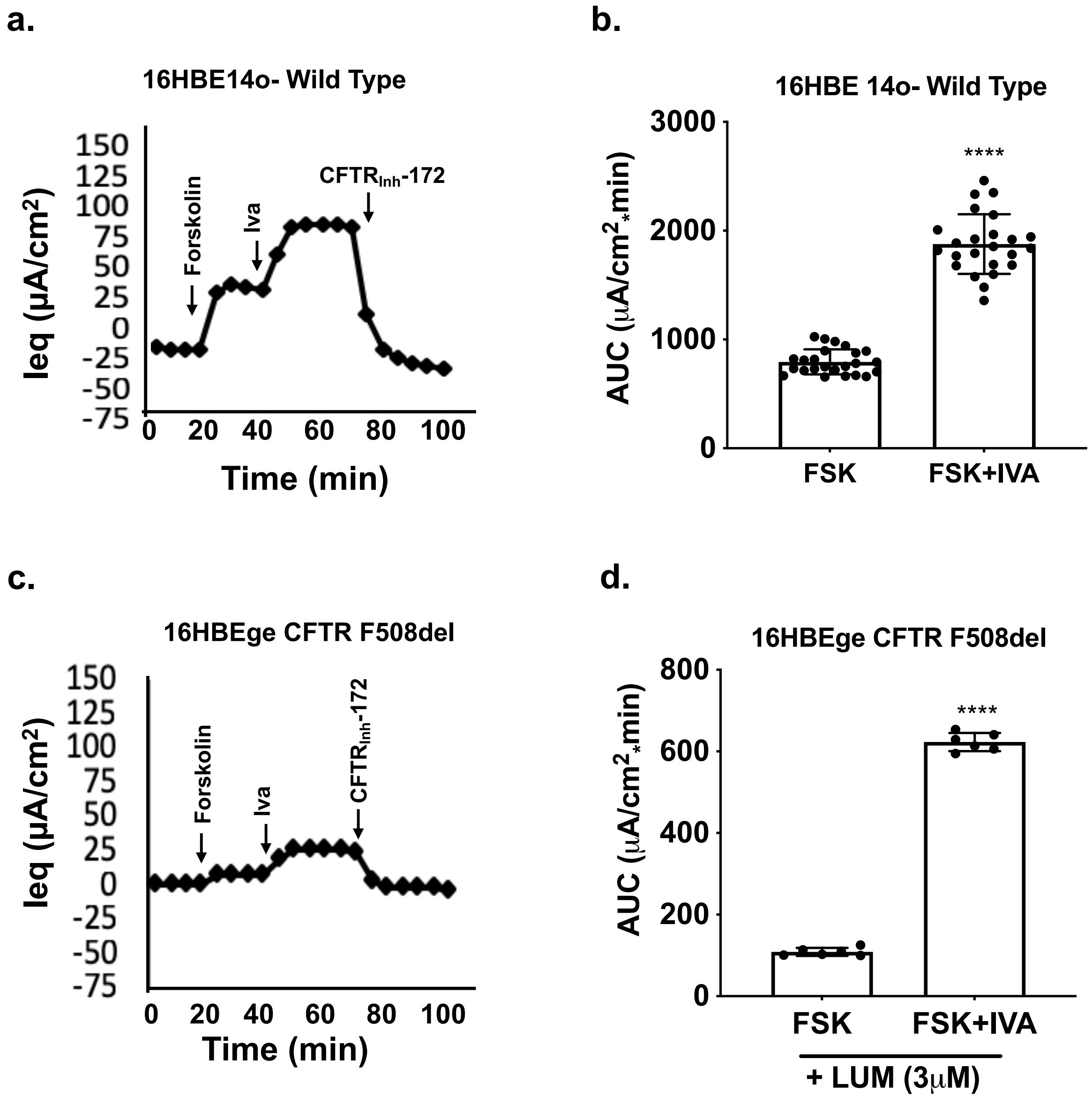

Suppl Fig. 6: CFTR activity in parental 16HBE14o- cells expressing wild-type CFTR and in gene-edited 16HBEge cells expressing CFTR F508del. CFTR activity measured in parental, wild-type 16HBE14o- cells as the change from baseline conductance following the acute addition of forskolin (10 μM) and ivacaftor (10 μM) followed by $CFTR_{Inh}$-172 (10 μM). **a.** Representative CFTR conductance tracings. **b.** Corresponding summary data representation of area under curve after forskolin plus ivacaftor stimulation to $CFTR_{Inh}$-172 inhibition (n=6, data are expressed as mean ± S.D. and statistically analyzed using unpaired t test, ****p<0.0001) **c, d.** CFTR activity in *CFTR*-F508del 16HBEge cells after 48 hours of treatment with lumacaftor (LUM, 3 μM). **c.** Representative CFTR conductance traces. **d.** Corresponding summary data (n=24, data are expressed as mean ± S.D. and statistically analyzed using unpaired t test, ****p<0.0001).. Each experiment was repeated at least twice with n=6-8 monolayers per condition during each replicate.

Supplementary Figure 7

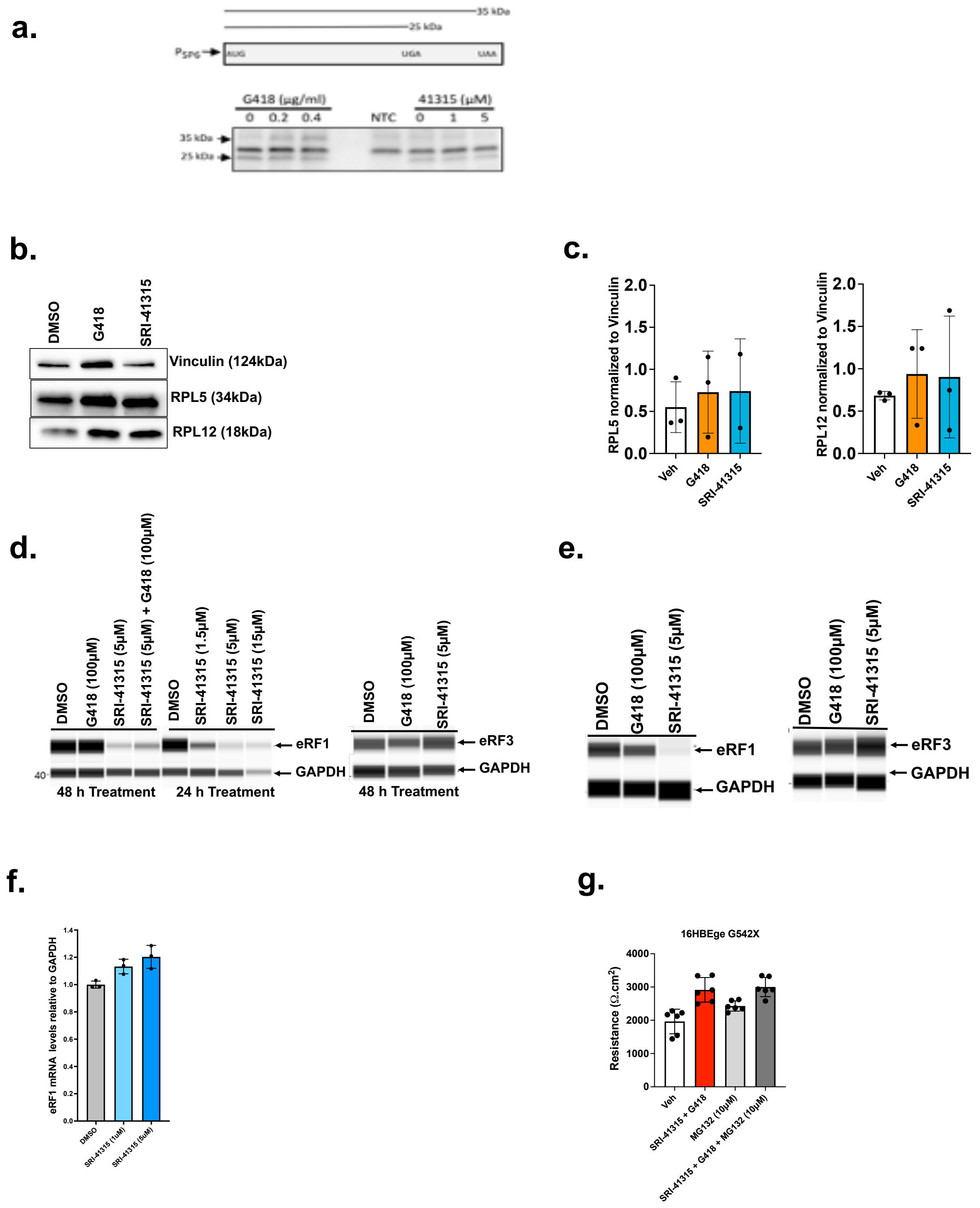

Supplementary Figure 7 (cont.)

h.

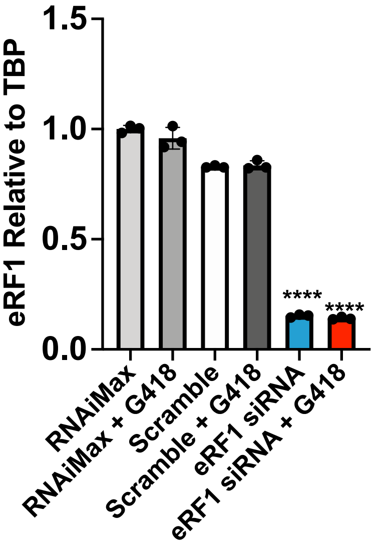

i.

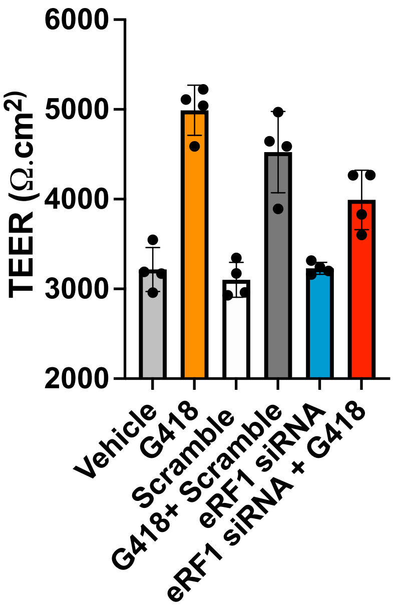

Suppl Fig. 7: SRI-41315 promotes depletion of eRF1 protein.

a. A readthrough reporter was expressed in a cell-free rabbit reticulocyte lysate *in vitro* transcription/translation system. A 25kDa protein was generated if translation terminated at the PTC and a 35kDa protein was generated if readthrough of the PTC occurred. A representative SDS-PAGE gel shows the protein products generated in *in vitro* transcription/translation reactions incubated with either G418 or SRI-41315. **b.** Representative western blot showing RPL5, RPL12 and the vinculin control after 48-hour treatment in *CFTR*-G542X 16HBEge cells. **c.** Corresponding quantification of western blots for RPL5 (left) and RPL12 (right) relative to vinculin from promoter-enhanced *CFTR*-G542X 6HBEge cells (corresponding to Fig. 6) and *CFTR*-G542X 16HBEge cells. Mean of at least 3 experiments per blot shown. **d.** Representative capillary western blot (Wes system, ProteinSimple) showing dose-dependent effects of SRI-41315 (1.5 µM, 5 µM and 15 µM) and/or G418 on eRF1 protein levels (left) or eRF3 levels (right) after 24 or 48 hours of treatment in promoter-enhanced *CFTR*-G542X 16HBEge cells. **e.** Representative “Wes” western blots in primary homozygous *CFTR*-W1282X HBE cells of eRF1 and eRF3 levels after SRI-41315 (5µM), G418 (100 µM), or DMSO control treatments for 48 hours. **f.** *eRF1* transcript levels determined by real time qRT-PCR relative to *GAPDH* after SRI-41315 and DMSO control treatments for 48 hours. **g.** Transepithelial resistances at baseline of *CFTR*-G542X 16HBEge cells treated with the indicated treatment conditions (functional data shown in Fig. 6e-f). **h.** eRF1 transcript levels determined by real time RT-PCR relative to TATA-box binding protein

(TBP) after transfection with either a scrambled siRNA control or an eRF1 siRNA to mediate its knockdown (96 hours) +/- G418 (total 72 hours with G418 replenishment at 48 hours). **i.** Transepithelial resistances at baseline of *CFTR*-G542X 16HBEge cells treated with the indicated treatment conditions (functional data shown in Fig. 6i-j).

Supplementary Figure 8

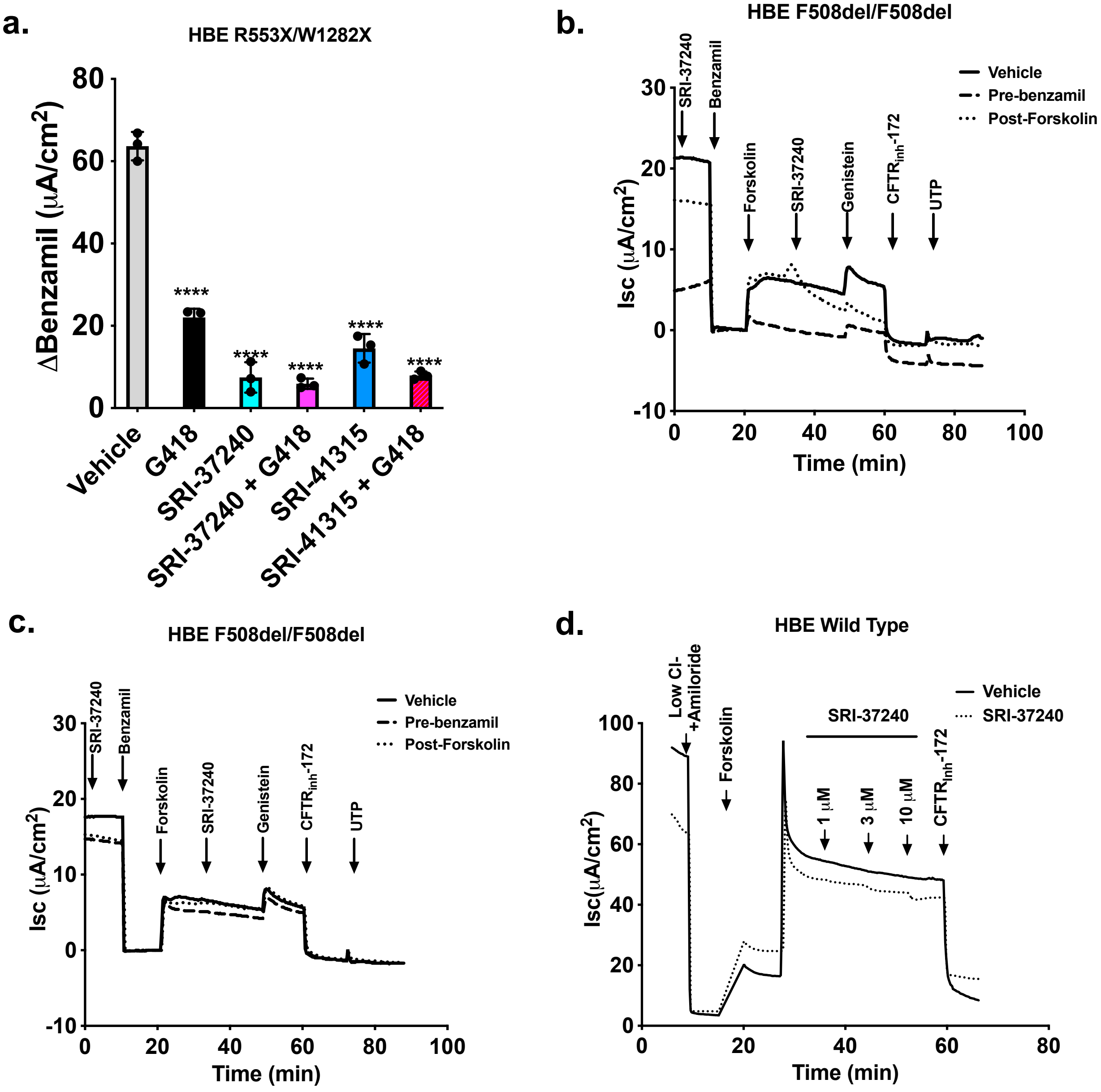

Suppl Fig. 8: Off-target effects of SRI-37240 and SRI-41315 on other ion channels.

Primary HBE cells derived from a R553X/W1282X donor were treated with SRI-37240 (10 µM), SRI-41315 (5 µM) or G418 (100 µM) for 72 hours. **a.** Bar graph illustrating the reduction of benzamil (6 µM) sensitive equivalent currents in response to a 72-hour treatment with SRI-37240 (10 µM), SRI-41315 (5 µM) and/or G418 (100 µM). Bars represent the average of three measurements exemplified in Fig. 7a (n=3, data are expressed as mean ± S.D. and statistically analyzed using Ordinary One-way ANOVA followed by Tukey's post-hoc test, ****p<0.0001). **b, c.** Representative Ussing tracings from HBE cells derived from a donor homozygous for *CFTR*-F508del treated for 48 hours with lumacaftor (3 µM). SRI-37240 (10 µM) was added acutely before benzamil or after forskolin addition, as indicated, as compared to vehicle. **c.** Similar experiment as B, but with SRI-37240 added to the basolateral bath solution (10 µM). **d.** Representative Ussing tracings from normal (non-CF) HBE cells. After stimulation with forskolin, SRI-37240 was added acutely in a dose dependent manner (1, 3 and 10 µM) to the apical bath solution (dotted trace) compared to vehicle (solid trace). n=3 monolayers per condition with 2-3 experimental repeats.

Supplementary References

1. Xue, X., *et al.* Identification of the Amino Acids Inserted During Suppression of CFTR Nonsense Mutations and Determination of Their Functional Consequences. *Hum Mol Genet* **26**, 3116-3129 (2017).
2. Habib, A.R., *et al.* A Systematic Review of the Clinical Efficacy and Safety of CFTR Modulators in Cystic Fibrosis. *Sci Rep* **9**, 7234 (2019).
3. Mutyam, V., *et al.* Therapeutic benefit observed with the CFTR potentiator, ivacaftor, in a CF patient homozygous for the W1282X CFTR nonsense mutation. *J Cyst Fibros* **16**, 24-29 (2017).
4. McDonald, C.M., *et al.* Ataluren in patients with nonsense mutation Duchenne muscular dystrophy (ACT DMD): a multicentre, randomised, double-blind, placebo-controlled, phase 3 trial. *Lancet* (2017).
