# Supplementary material for: A small molecule that induces translational readthrough of CFTR nonsense mutations by eRF1 depletion
Source: Nat Commun. 2021 Jul 16;12:4358. doi: 10.1038/s41467-021-24575-x (PMC8285393; doi:10.1038/s41467-021-24575-x)
Supplement: Supplementary file 2 — Reporting Summary [file 41467_2021_24575_MOESM2_ESM.pdf]

nature research

Corresponding author(s): David Bedwell

Last updated by author(s): Mar 3, 2021

Reporting Summary

Nature Research wishes to improve the reproducibility of the work that we publish. This form provides structure for consistency and transparency in reporting. For further information on Nature Research policies, see our Editorial Policies and the Editorial Policy Checklist.

Statistics

For all statistical analyses, confirm that the following items are present in the figure legend, table legend, main text, or Methods section.

n/a	Confirmed	
[ ]	[x]	The exact sample size (n) for each experimental group/condition, given as a discrete number and unit of measurement
[ ]	[x]	A statement on whether measurements were taken from distinct samples or whether the same sample was measured repeatedly
[ ]	[x]	The statistical test(s) used AND whether they are one- or two-sided<br>*Only common tests should be described solely by name; describe more complex techniques in the Methods section.*
[x]	[ ]	A description of all covariates tested
[x]	[ ]	A description of any assumptions or corrections, such as tests of normality and adjustment for multiple comparisons
[ ]	[x]	A full description of the statistical parameters including central tendency (e.g. means) or other basic estimates (e.g. regression coefficient) AND variation (e.g. standard deviation) or associated estimates of uncertainty (e.g. confidence intervals)
[ ]	[x]	For null hypothesis testing, the test statistic (e.g. F, t, r) with confidence intervals, effect sizes, degrees of freedom and P value noted<br>*Give P values as exact values whenever suitable.*
[x]	[ ]	For Bayesian analysis, information on the choice of priors and Markov chain Monte Carlo settings
[x]	[ ]	For hierarchical and complex designs, identification of the appropriate level for tests and full reporting of outcomes
[x]	[ ]	Estimates of effect sizes (e.g. Cohen's d, Pearson's r), indicating how they were calculated

Our web collection on statistics for biologists contains articles on many of the points above.

Software and code

Policy information about availability of computer code

Data collection	GloMax Assay Detection System (Bio-Rad) was used to collect luciferase and spectrophometric data; GelDocXR+Gel Documentation System (Bio-Rad), ChemiDocXRS+ Imaging System (Bio-Rad), and Wes (ProteinSimple) were used to collect and visualize western blotting data; CFTR functional data was collect using transepithelial current clamp (TECC-24) amplifier (EP Design BVBA) and Ussing Chamber, NanoDrop spectrophotometer was used to determine DNA/RNA concentrations (Thermofisher)
Data analysis	Xlfit Module ActivityBase software was used to calculate CC50 values for HTS data. GraphPad Prism v5 software (GraphPad Software, Inc.) was used to perform statistics. Seqtk, Skewer, STAR, GENCODE, and Python 2.7 were all used to analyze ribosomal profiling data. ChemiDoc XRS+ Gel Documentation software and Compass software (ProteinSimple) were used to analyze western blotting data.

For manuscripts utilizing custom algorithms or software that are central to the research but not yet described in published literature, software must be made available to editors and reviewers. We strongly encourage code deposition in a community repository (e.g. GitHub). See the Nature Research guidelines for submitting code & software for further information.

Data

Policy information about availability of data

All manuscripts must include a data availability statement. This statement should provide the following information, where applicable:

- Accession codes, unique identifiers, or web links for publicly available datasets
- A list of figures that have associated raw data
- A description of any restrictions on data availability

All relevant data are available from the authors. Source data are provided with this paper.

Field-specific reporting

Please select the one below that is the best fit for your research. If you are not sure, read the appropriate sections before making your selection.

☒ Life sciences ☐ Behavioural & social sciences ☐ Ecological, evolutionary & environmental sciences

For a reference copy of the document with all sections, see nature.com/documents/nr-reporting-summary-flat.pdf

Life sciences study design

All studies must disclose on these points even when the disclosure is negative.

Sample size	Many of the assay parameters were previously determined and their references are cited in the manuscript where appropriate. Assays for CFTR function have been widely used, and the SD is known; replicates were conducted based on that experience. For luminescent/fluorescent reporters, we evaluated the SD and signal to background, then chose sufficient replicates to determine a reasonable difference. Reproducibility was high and SNR large, therefore the number of replicates were chosen for technical validity. All Western blots were repeated 3 times for the proteins of interest. The biological sample size was determined by the experimental variability and the variability between biological sample as well as the number of samples required to perform statistical analysis.
Data exclusions	No data was excluded from the study unless specifically stated and the statistical method chosen to determine which flyer points were dropped.
Replication	Each experiment was performed with at least two replicates. The replicate number for each experiment is now indicated in the figure legends. All attempts at replication were successful.
Randomization	Cell culture studies were used with homogeneous replicates where randomization would not improve the experimental design or analysis. Plate positioning of controls and experimental conditions were altered through experimental replicates to assure technical homogeneity.
Blinding	Compounds were advanced for cell culture testing without communication of which structures and chemical series were represented. Analytical methods included standardized methods for determining valid experimental replicates to remove bias.

Reporting for specific materials, systems and methods

We require information from authors about some types of materials, experimental systems and methods used in many studies. Here, indicate whether each material, system or method listed is relevant to your study. If you are not sure if a list item applies to your research, read the appropriate section before selecting a response.

Materials & experimental systems

n/a	Involved in the study	
☐	☒	Antibodies
☐	☒	Eukaryotic cell lines
☒	☐	Palaeontology and archaeology
☒	☐	Animals and other organisms
☒	☐	Human research participants
☒	☐	Clinical data
☒	☐	Dual use research of concern

Methods

n/a	Involved in the study	
☒	☐	ChIP-seq
☒	☐	Flow cytometry
☒	☐	MRI-based neuroimaging

Antibodies

Antibodies used	Antibodies used include: Monoclonal CFTR primary antibody (UNC 596, clone IgG2b, lot 596TJ20140318 received under MTA from the University of North Carolina, at Chapel Hill), a-tubulin (Thermo Fisher #62204, clone T5168, lot N.A.), N-terminus of eRFI (Cell Signaling #13916, polyclonal, lot 1), C-terminus of eRFI (Santa Cruz #sc-365686, B-11, E2412), eRF3 (Cell Signaling #14980, polyclonal, lot 1), RPL5 (Cell Signaling #14568, polyclonal, lot 1), RPL12 (Abcam #ab157130, polyclonal, lot GR175991-9), eIF5A (Cell Signaling #20765, clone D8L8Q, lot 1), UPF2 (Cell Signaling #11875, clone D3B10, lot 1), SMG6 (Abcam #ab87539, polyclonal, lot GR201509-1), GAPDH (Cell Signaling #2118, clone 14C10, lot 10), vinculin (Cell Signaling #13901, clone E1E9V, lot 2), anti-Rabbit IgG (H+L) HRP (Jackson Immunoresearch #111-035-144, whole IgG affinity-purified, lot 130174), and anti-mouse IgG (H+L) HRP (Jackson Immunoresearch #115-035-166, whole IgG affinity-purified, lot 139034). Please note that the dilution used for each antibody is listed in the Materials & Methods.
Validation	Monoclonal CFTR primary antibody (UNC 596 received under MTA from the University of North Carolina, at Chapel Hill) is extensively used in the CF field. The following antibodies were validated by the vendor (additional information is available at the manufacturer links provided): a-tubulin (Thermo Fisher #62204, https://www.thermofisher.com/antibody/product/alpha-Tubulin-Antibody-clone-DM1A-Monoclonal/62204), N-terminus of eRFI (Cell Signaling #13916, https://www.cellsignal.com/products/primary-antibodies/erf1-antibody/13916), C-terminus of eRFI (Santa Cruz #sc-365686, https://www.scbt.com/p/erf1-antibody-b-11), eRF3 (Cell Signaling #14980, https://www.cellsignal.com/products/primary-antibodies/erf3-antibody/14980), RPL5 (Cell Signaling #14568, https://

www.cellsignal.com/products/primary-antibodies/rpl5-antibody/14568), RPL12 (Abcam #ab157130, https://www.abcam.com/rpl12-antibody-ab157130.html), eIF5A (Cell Signaling #20765, https://www.cellsignal.com/products/primary-antibodies/eif5a-d8l8q-rabbit-mab/20765), UPF2 (Cell Signaling #11875, https://www.cellsignal.com/products/primary-antibodies/upf2-d3b10-rabbit-mab/11875), SMG6 (Abcam #ab87539, https://www.abcam.com/smg6-antibody-ab87539.html), GAPDH (Cell Signaling #2118, https://www.cellsignal.com/products/primary-antibodies/gapdh-14c10-rabbit-mab/2118?site-search-type=Products&N=4294956287&Ntt=14c10&fromPage=plp), vinculin (Cell Signaling #13901, https://www.cellsignal.com/products/primary-antibodies/vinculin-e1e9v-xp-rabbit-mab/13901), anti-Rabbit IgG (H+L) HRP (Jackson Immunoresearch #111-035-144, https://www.jacksonimmuno.com/catalog/products/111-035-144), and anti-mouse IgG (H+L) HRP, (Jackson Immunoresearch #115-035-166, https://www.jacksonimmuno.com/catalog/products/115-035-166). In addition, knock-downs of eRFI in our study (Fig. 6G) further validated the specificity of the eRFI antibody.

Eukaryotic cell lines

Policy information about cell lines

Cell line source(s)	HEK293 (ATCC); Fischer rat thyroid and human bronchial epithelia (CFFT lab)
Authentication	Each human cell line was authenticated using a 16 STR marker profile; each rat cell line was authenticated using a 31 STR marker profile. The genetic profiles were performed by Idexx Bioanalytics. No evidence of cross contamination was found.
Mycoplasma contamination	All cell lines were tested by Idexx Bioanalytics and found to be negative for mycoplasma.
Commonly misidentified lines (See ICLAC register)	None.
